# Bacterial Infections in Hematopoietic Stem Cell Transplant Recipients

**DOI:** 10.4084/MJHID.2015.045

**Published:** 2015-07-01

**Authors:** Elisa Balletto, Małgorzata Mikulska

**Affiliations:** Division of Infectious Diseases, IRCCS San Martino University Hospital – IST, Genoa, Italy. Department of Health Sciences, University of Genoa, Genoa, Italy

## Abstract

Bacterial infections are major complications after Hematopoietic Stem Cell Transplant (HSCT). They consist mainly of bloodstream infections (BSI), followed by pneumonia and gastrointestinal infections, including typhlitis and *Clostridium difficile* infection. Microbiological data come mostly from BSI. Coagulase negative staphylococci and Enterobacteriaceae are the most frequent pathogens causing approximately 25% of BSI each, followed by enterococci, *P. aeruginosa* and viridans streptococci. Bacterial pneumonia is frequent after HSCT, and Gram-negatives are predominant. *Clostridium difficile* infection affects approximately 15% of HSCT recipients, being more frequent in case of allogeneic than autologous HSCT.

The epidemiology and the prevalence of resistant strains vary significantly between transplant centres. In some regions, multi-drug resistant (MDR) Gram-negative rods are increasingly frequent. In others, vancomycin-resistant enterococci are predominant. In the era of increasing resistance to antibiotics, the efficacy of fluoroquinolone prophylaxis and standard treatment of febrile neutropenia have been questioned. Therefore, a thorough evaluation of local epidemiology is mandatory to decide the need for prophylaxis and the choice of the best regimen for empirical treatment of febrile neutropenia. For the latter, individualised approach has been proposed, consisting of either escalation or de-escalation strategy. De-escalation strategy is recommended since resistant bacteria should be covered upfront, mainly in patients with severe clinical presentation and previous infection or colonisation with a resistant pathogen.

Non-pharmacological interventions, such as screening for resistant bacteria, applying isolation and contact precautions should be put in place to limit the spread of MDR bacteria. Antimicrobial stewardship program should be implemented in transplant centres.

## Introduction

Bacterial infections are among the major complications of hematopoietic stem cell transplant (HSCT). The most frequent clinical entities are bloodstream infections (BSI), pneumonia and gastrointestinal infections, which include typhlitis and infections due to *Clostridium difficile*. Infections due to Gram-negative rods used to be the main cause of infection-related mortality during neutropenia. Fortunately, over the decades numerous successful strategies have been developed to limit the negative impact of these infections. In fact, with the universal use of prompt empirical antibiotic therapy in case of fever during neutropenia and, in some settings, antibiotic prophylaxis, the fatality rate dropped significantly.^1^

However, the recent emergence and spread of multidrug-resistant (MDR) bacteria, particularly Gram-negatives, threaten to nullify all the progress made in the field of preventing and treating bacterial infections, since the pathogens that are resistant to all antimicrobials commonly used as empirical treatment are becoming more and more frequent in HSCT recipients worldwide.^2^,^3^

This review will focus on recent changes in the epidemiology of bacterial infections, mostly BSI, after HSCT, highlighting the epidemiology of MDR pathogens such as methicillin-resistant staphylococci, vancomycin resistant enterococci (VRE), Enterobacteriaceae producing extended-spectrum beta-lactamases (ESBLs), MDR Enterobacteriaceae, mostly carbapenemase-producing *K. pneumoniae* (KPC - *K. pneumoniae*) and MDR *P. aeruginosa*. The epidemiology of *C. difficile* infections will be briefly reviewed. Additionally, advances in the management of the MDR infections, such as a new approach to empirical therapy and antimicrobial stewardship will be discussed.

### Epidemiology

The most common bacterial infections after HSCT are BSI, pneumonia and gastrointestinal infections. Urinary tract infections are infrequent and usually associated with the presence of the urinary catheter. The reliable data on the aetiology of bacterial infections in the setting of HSCT come mainly from the results of blood cultures. In fact BSI is the most frequent microbiologically documented infection, whereas microbiological documentation is significantly less frequent in case of pneumonia or typhlitis.

## Bloodstream infections

BSI affects approximately 5–10% of autologous and 20–30% of allogeneic HSCT recipients, with significant variations between centres and between patients undergoing different transplantation procedures, e.g. type of conditioning regimen. The incidence of BSI is the highest during the pre-engraftment neutropenia and depends mainly on the extent of oral and enteric mucositis and the presence of a central venous catheter. During later non-neutropenic phases, BSIs are more frequent in case of Graft-versus-Host-Disease (GvHD), the presence of hypoglobulineamia or central venous catheter. The main risk factors associated with BSI due to single bacterial species are reported in Table 1.

Following a growing body of data on the emergence of resistant Gram-negative rods, 4th European Conference on the Infections of Leukemia (ECIL-4) in 2011 addressed the issue of bacterial infections in this setting. In order to understand the extent of the problem, a review of the literature was performed and a questionnaire was sent to participating centres focusing of the current epidemiology, resistant patters and recommended empirical therapy.^4^ Additionally, a prospective observational study on Gram-negative BSI in HSCT recipients is ongoing (registered as ClinicalTrials.gov Identifier: NCT02257931).

The review of the literature published after 2005 yielded 29 reports from 13 countries concerning autologous (14 reports) and allogeneic (19 reports) HSCT.^4^ The median year of observation was 2001, ranging from 1987 to 2009. The Gram-positive to Gram-negative ratio was 60% vs. 40%, respectively, with some centres reporting the ratio of 85% vs. 15%, while, others 26% vs. 74%. The ECIL-4 questionnaire included answers from 33 centres from 18 countries (autologous HSCT in 32 and allogeneic HSCT in 30 centres), with the median year of observation of 2008 (range, 1998–2010). These more recent data indicated a further decrease in Gram-positive to Gram-negative ratio (55% vs. 45%), with similar huge differences between the centres from 85% vs. 15% in some to 30% vs. 70% in others.

**Figure 1:**
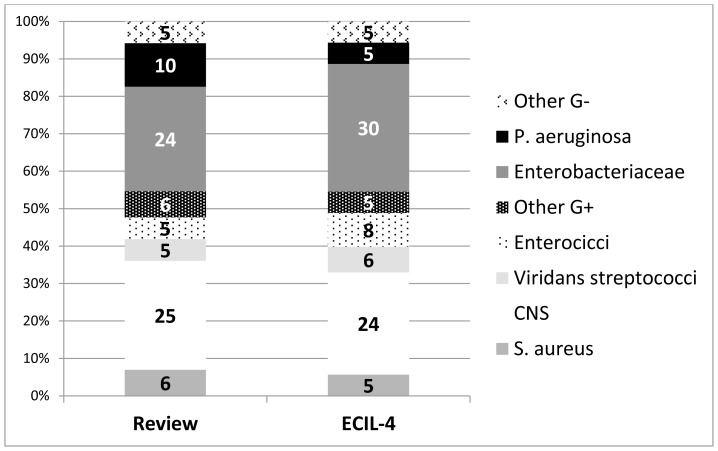
The aetiology of bloodstream infections according to literature review and questionnaire survey performed for European Conference on Infections in Leukemia (ECIL), reported as median values.^4^

More in detail, Enterobacteriaceae and coagulase-negative staphylococci were the most frequently isolated pathogens (Figure 1). Compared to published data, in the ECIl-4 questionnaire the incidence of *P. aeruginosa* was lower, but the incidence of enterococci was higher.^4^

### Staphylococci

Staphylococci are the most frequent pathogens isolated during BSI. They are mostly coagulase-negative (approx. 25 % of all BSI), while *S. aureus*, a species significantly more virulent, is associated only with smaller proportion of infections (approx. 5%).^4^ This high rate might be in part explained by the fact that not all the studies and centres regarded coagulase-negative staphylococci as a true cause of BSI only if isolated in two consecutive blood cultures.

As far as resistance pattern is concerned, in ECIL centres more than half of isolated coagulase-negative staphylococci were resistant to methicillin while the rate of methicillin-resistance in *S. aureus* has been reported lower.^4^ In the literature review, methicillin-resistance was also more frequent among coagulase-negative staphylococci than *S. aureus*, with respective median resistance rates of 80% and 56%.^4^ Of note, the resistance to methicillin has been reported lower in children than in the adult population.

Although the overall incidence of methicillin-resistant *S. aureus* (MRSA) BSI is low in HSCT setting, concerns about increased mortality have been raised. In particular, in two cases of MRSA outbreak, the attributable mortality was very high. In the UK outbreak, it was probably over 20% while in an Australian outbreak in 41 neutropenic patients, the attributable fatality rate was 50%.^5^,^6^ Hopefully, outside an outbreak setting, the outcome of MRSA infections is more favourable, particularly in centres where methicillin-resistant staphylococci are regularly seen, and glycopeptides are frequently used in empirical therapy. Infection control measures, found effective against MRSA, include alcohol-based hand hygiene, nasal screening, universal or selective decolonization, improvement in central line management, and a reduction in the use of fluoroquinolones, and are all currently recommended by international guidelines.^7^

Good news concerning MRSA infections is that, for reasons that remain yet to be fully investigated, since 2004 a worldwide confirmed decline in MRSA has been noted in the US, and in several European and Far East countries, despite different infection-control approaches undertaken.^8^,^9^

Finally, several new therapeutic options active against MRSA have been introduced in the last five years, including anti-MRSA cephalosporins such as ceftaroline or ceftobiprole, lipoglycopeptides such as telavancin, dalbavancin or oritavancin, or a new oxazolidinone: tedizolid.^10^ Although none of these drugs has been approved for empirical or targeted treatment of infections in neutropenic patients, they offer much needed alternatives for better management of methicillin-resistant infections. Among them, cephalosporins might be particularly attractive due to their historically known efficacy and safety while some novel lipoglycopeptides might revolutionise outpatient treatment allowing for once weekly administration.

### Enterobacteriaceae

Enterobacteriaceae, and in particular *E. coli*, are the second most common pathogen in BSI, being only slightly less frequent than staphylococci. The mortality associated with infections due to Enterobacteriaceae is directly associated with the time to the onset of an effective antibacterial therapy. As demonstrated in the comparison between ESBL-positive and ESBL-negative BSIs due to *E. coli*, the time to the appropriate empirical therapy was longer, and the outcome was poorer, in case of ESBL-producing strains.^11^–^13^

In most of the European countries, over 10% of all invasive infections caused by *E. coli* in 2012 were due strains unsusceptible to 3rd generation cephalosporins and the prevalence of ESBL producing strains in patients with haematological malignancies varies, being for example 13% in Spain and 48% in Japan.^11^,^14^ The ECIl-4 literature review reported that in median 34% of Enterobacteriaceae were ESBL-positive, ranging from 16% to 44% in different centres; whereas according to ECIL-4 questionnaire over 60% of centres reported that only less than 25% of Enterobacteriaceae were ESBL-producers, including 20% of centres with the prevalence of ESBL-producers of < 5%.^4^

In another experience from Spain in patients with haematological malignancies, MDR Gram-negatives (including ESBL-producing strains) represented 11% of all Gram-negatives, and a significant increase has been observed compared to the previous observation period (11% vs. 3%).^15^,^16^

Carbapenem-resistant Enterobacteriaceae are the most recent and rapidly spreading threat, and in Europe they consist mainly of carbapenem-resistant *K. pneumoniae*. In fact, in 29 European countries the mean incidence of carbapenem resistance in *K. pneu*moniae was 6%, ranging from 0 to 61%,^8^ and single-centre outbreaks and national epidemics have been reported in Greece and Italy, which are now considered endemic for KPC - *K. pneumoniae*.^8^,^17^ Until recently, few reports focused exclusively on patients with hematologic malignancy and KPC - *K. pneumoniae* BSI, but the reported attributable mortality rates were 38%, 56% and 67%.^17^ Therefore, multidisciplinary intensive programs that address the issue of limiting the spread of these bacteria are warranted.

Last but not least, the issue of resistance of Gram-negatives to fluoroquinolones is worrisome. Interestingly, in several centres, the rate of fluoroquinolone resistance in *E. coli* increased irrespectively of the use of prophylaxis by the transplant centre. For example in Sweden, despite the absence of fluoroquinolone prophylaxis, the resistance in *E. coli* increased significantly from 2% in years 1995–2001 to 16% in 2002–2008.^18^ In Japan, there were no *E. coli* resistant to fluoroquinolones during the years 2003–2005 when the prophylaxis was in place, but in years 2006–2009, when fluoroquinolone prophylaxis was not prescribed, over 60% of *E. coli* tested were resistant.^19^ These results might reflect a worldwide trend in the general increase in fluoroquinolone resistance in Enterobacteriaceae.^8^ Since fluoroquinolone prophylaxis is recommended and widely used in neutropenic adults receiving allogeneic HSCT, it is not recommended for empirical treatment of febrile neutropenia.^1^,^20^ Thus, the rate of fluoroquinolone resistance among Gram-negatives does not influence significantly therapeutic choices, but it may have severe implications for the prophylactic strategy in neutropenic HSCT recipients. In fact, the benefit of fluoroquinolone prophylaxis is considered uncertain when the prevalence of fluoroquinolone-resistance in Gram-negative rods exceeds 20%.^21^ Therefore, abolishing any antibiotic prophylaxis might be reasonable in the era of multidrug resistance, despite the fact that an increase in Gram-negative BSI was observed in some centres where prophylaxis was discontinued.^14^,^15^

### Enterococci

Enterococci have emerged as the third most frequent group of bacterial pathogens in BSI, affecting even 10%–12% of all transplant patients.^22^–^26^

Compared to other pathogens, enteroccocal BSI usually occurs later after transplant, for example, the median day for pre-engraftment BSI was day +4 for viridans and +11 for enterococci.^27^ In many centres, *E. faecium* almost completely replaced *E. faecalis*, with important therapeutic consequences since *E. faecium* is frequently resistant to ampicillin.^24^,^28^,^29^

In some centres, the shift from *E. faecalis* to *E. faecium* has been also accompanied by an important increase in the rate of resistance to vancomycin. In a multicentre Australian study VRE increased from approximately 8% in 2001–2004 period to 64% in years 2007–2010.^24^ The problem of vancomycin-resistance is important in HSCT recipients since few therapeutic options are available, and high mortality in patients infected with VRE has been reported.^30^,^31^ In general, there is a low incidence of VRE in European centres with less than 5% of enterococci, being VRE in 67% haematology centres in the ECIL-4 questionnaire, in accordance with the general European data reporting low prevalence of VRE in most countries in Western Europe.^4^,^8^,^22^,^27^–^29^ On the contrary, in the US up to 80% of *E. faecium* are VRE.^25^,^26^,^30^ In fact, these are mostly the reports from the US centers that highlight an important mortality in patients with VRE infection.

However, it remains debatable if the resistance to vancomycin is to blame for this poor outcome. In fact, enterococci are low virulence pathogens and numerous concomitant clinical problems are usually present in patients with enterococcal BSI.^32^ Moreover, evaluating the directly attributable mortality of enteroccocal sepsis in patients with multiple clinical problems is subjective, and arbitrary even if universally high; furthermore the 30-day overall mortality might simply indicate that VRE could be a marker of clinical severity.^25^,^26^,^29^,^30^,^33^

This view is supported by several clinical experiences. In one study, a delayed use of adequate antibiotics in case of VRE infection resulted in no difference in 30-day mortality compared to vancomycin-susceptible infections in neutropenic patients, and only underlying severity of medical condition predicted outcome.^34^ In another study, Brasilian authors found that empirical treatment of neutropenic fever with linezolid had no effect on survival (54% vs. 42%) in 100 haematology patients who were colonised with VRE, while the mortality was associated only with the persistence of neutropenia and GvHD.^35^

Finally, in our experience in a cohort of 67 adult allogenic HSCT recipients with enterococcal BSI, of whom only 13% had VRE infection, 30-day mortality for vancomycin-susceptible and VRE was respectively, 26% and 11%, whereas 1-year overall survival was 24% for both goups compared to 65% in patients with no enterococcal BSI.^36^ These results were compared with an experience of a US transplant center, where 66% of patients with enterococcal BSI had VRE; 30-day mortality was 38% for both vancomycin-susceptible and resistant enterococci; while 1-year overall survival was 48% for vancomycin-susceptible enterococci, 23% for VRE and 63% for patients with no enterococcal BSI.^37^

Treatment of VRE is based on the use of linezolid, for which satisfactory efficacy data in this setting have been reported. Of note, hematologic side effects, which are particularly important in HSCT recipients, have not been reported significant; in particular time to neutrophil and platelet engraftment has been not found different in 33 cases who received more than 7 days of linezolid treatment during pre-engraftment phase, compared to controls.^38^ Resistance of enterococci to linezolid is rare and usually mediated by mutations 23S rRNA target.^39^ It has been associated with previous linezolid therapy, although nosocomial acquisition of resistant enterococci has been also reported.^39^–^41^ Resistance mechanisms were first described for *E. faecium* and *S. aureus*, and later also for *E. faecalis*, but they remain rare, affecting less than 1% of all strains, as documented in a surveillance study of 7608 clinical isolates of enterococci from years 2004–2012 collected in the USA.^42^

Daptomycin, for which in vitro activity has been documented but clinical data are limited in HSCT setting, is another important therapeutic option against VRE.^43^ Several meta-analyses and systematic reviews have addressed the comparison of outcomes of VRE BSI treated with linezolid and daptomycin.^44^–^46^ With the evident limit of the low quality of studies included (mostly retrospective, no randomised trials), the mortality rates were found slightly higher in case of daptomycin, compared to linezolid.^44^–^46^

Other options are quinopristin-dalfopristin, which is active only against *E. faecium*, and not *E. faecalis*, and tigecycline, with the well-known limit of low blood levels.^47^ Novel cephalosporins seem inactive against enterococci while novel glycolipopetides such as telavancin and dalbavancin seem active only against some (VanB) strains.

In conclusion, enterococci are increasingly frequent in HSCT setting, *E. faecium* is the predominant species, but resistance to vancomycin varies significantly between geographical regions. Enterococcal infections, both due to VRE and vancomycin-susceptible *E. faecium*, could be regarded as a marker of poor clinical status and important comorbidities.^29^

### Pseudomonas aeruginosa

*Pseudomonas aeruginosa* is a Gram-negative pathogen traditionally associated with the highest mortality rate, both during neutropenia and later after HSCT. Fortunately, currently its prevalence in infections of European haematology centres is lower than reported in published reports (respectively, 5% and 10%), although in some centres it may cause up to 30% of all BSI.^4^ Along with high virulence, *P. aeruginosa* is characterised by numerous intrinsic or acquired resistance mechanisms, including adaptive mechanisms, which make numerous antibiotic options ineffective.^48^ In particular, it is characterised by high intrinsic resistance due to low outer membrane permeability, which limits antibiotic penetration, beta-lactamase production and efflux pump overexpression. Additionally, adaptive resistance mechanisms such as genes expression changes lead to further efflux increase and enzyme production. Finally, intrinsic mechanisms can be potentiated by acquired resistance mechanisms which include single or numerous mutations, or, less frequently, horizontal transfer of resistance determinants leading to reduced uptake and efflux pump overexpression.^48^

In fact, the resistance to carbapenems is high, with the mean value in Europe of 17%, and national estimates between 3% and 51%.^8^ In a multicentre Italian experience from years 2009–2010, 71% of *P. aeruginosa* strains causing BSI were MDR, with 60% of them being resistant to carbapenems.^49^ The 30-day mortality was clearly associated with the resistance: 40% for MDR strains and 9% for susceptible ones.^49^ Similar high resistance rates were reported in India, where 77% of *Pseudomonas* strains were MDR.^50^

Although most of the cases of MDR *P. aeruginosa* infections in HSCT recipients stem from *in vivo* induction of resistance mechanisms, outbreaks of *P. aeruginosa* infections have been reported in HSCT units.^51^,^52^ These outbreaks, similarly to that occurring in other settings, in particular adult or neonatal intensive care units (ICU), might have environmental source of infections (e.g. devices, soap or cleaning solutions, etc.), and might be long lasting, difficult to control and burdened with high morbidity and mortality.^51^–^54^ Along with outbreaks documenting the clonal origin of the infective strains, outbreaks not originating from a common source warrant attention since *P. aeruginosa* may be a water-borne pathogen; thus, such outbreaks may be associated with breaches in proper management of central venous catheters.^55^,^56^ European guidelines for the management of the infection control measures to reduce transmission of multidrug-resistant Gram-negative bacteria in hospitalized patients have been recently published.^57^

Colistin remains the cornerstone of the treatment of MDR *P. aeruginosa*, with the uncertainties concerning the optimal dosing, the need for combination therapy and the rate of toxicity.^58^

### Acinetobacter baumannii

*A. baumannii* is a non-fermentative coccobacillus that is widely distributed in nature and characterized by frequent MDR due to multiple mechanisms.^59^,^60^ Recently, BSI due to MDR *A. baumannii* has emerged as a major cause of health care-associated infections, especially in critically ill population, including immunocompromised patients.^61^ It is generally associated with a high crude mortality rate, ranging between 17% and 52%.^62^ Risk factors for infections with MDR *A. baumannii* in the immunocompromised include previous colonization, comorbid conditions, recent major surgical procedures, prolonged broad-spectrum antimicrobial therapy, prolonged hospitalization, admission to ICU and mechanical ventilation.^63^

Little is known regarding the incidence and risk factors for this infection in HSCT recipients.^60^,^64^ In the aforementioned ECIL-4 literature review and questionnaire, *A. baumannii* was responsible for a median of 2% of all BSIs, being absent in some centres but rising up to as high as 12% of all BSIs in others.^4^ In a retrospective case-control study Kim et al. found that the total incidence of MDR *A. baumannii* BSI was 0.52 cases/10,000 patient-days, with a mortality rate of 95%. The interval between admission and HSCT and a history of care in ICU after HSCT were independent risk factors for the development of *A. baumannii* infection.^65^ These features suggest that this infection affects predominantly patients who require intensive and invasive support, particularly ICU care and mechanical ventilation therapy after HSCT. Of note, in almost 90% of cases BSI developed after engraftment and lungs were the origin of infection in all the patients.^65^

Antimicrobial agents that are potentially effective against *A. baumannii* include carbapenems, beta-lactam inhibitors such as sulbactam, piperacillin–tazobactam and 3^rd^ generation cephalosporins. New options for MDR *A. baumannii* infections are old polypeptide antibiotics such as colistin or polymyxin B, minocycline derivatives such as tigecycline, new carbapenems such as doripenem, and new generation cephalosporins such as ceftobiprole and ceftaroline.^59^,^63^ In uncomplicated infections, the use of a single active beta-lactam may be justified, while definitive treatment of complicated infections in critically ill individuals may require drug combinations such as colistin and rifampicin or colistin and carbapenem.^60^

In conclusion, MDR *A. baumannii* BSI in HSCT recipients is a fatal infectious complication with no controlled trials to guide the therapeutic choices. As in case of others MDR pathogens, an approach which stratifies the risk of developing infection, and a prompt administration of active antimicrobial therapy, chosen on the basis of local epidemiology and previous colonization, may hopefully lead to better clinical outcomes.

### Viridans streptococci

Viridans streptococci have been traditionally associated with oral mucositis in course of chemotherapy (see Table 1). Although usually susceptible to beta-lactams, the risk of developing septic shock and acute distress respiratory syndrome (ARDS) has been reported as high, varying from 7% to 39%.^66^ Given high mortality rates reported in early studies, administration of corticosteroids to neutropenic patients with viridans streptococci BSI who develop early signs of respiratory failure have been studied with the aim of preventing the progression to ARDS and improving the survival.^67^–^69^

Nowadays, viridans streptococci are responsible for approximately 5% of all BSI. *Streptococcus mitis* is the most frequently isolated species, and it is also the species associated more frequently with resistance to penicillin and fluoroquinolones.^70^ The association between high penicillin MIC values, clinical outcome and the need for vancomycin treatment has been elegantly discussed in a recent editorial.^66^

## Pneumonia

Most of the studies describing infectious complications in HSCT patients show a high frequency of pneumonia,^71^–^73^ with an incidence reported in retrospectives studies ranging between 15% and 25%.^74^,^75^

Numerous acute pulmonary complications may occur in this population including both infectious and non-infectious causes, hence it is often difficult to obtain an aetiological diagnosis. The clinical setting and microbiological analyses, such as cultures of blood samples, sputum and bronchoalveolar lavage fluid, can be used to provide clues for interpreting abnormal CT finding but infections with more than one pathogens (e.g. bacterial and viral) and coexistence of infectious and non-infectious processes (e.g. viral and immunological) further hamper the precise description of epidemiology in this setting.

Therefore, the results of a nationwide prospective study referring to data collected by the Spanish Research Network of Transplant (RESITRA) are particularly interesting.^76^ From July 2003 to April 2005 427 HSCT recipients were followed with standardized diagnostic protocol for pneumonia. There were 112 episodes of pneumonia and 72 (64%) of them were microbiologically defined. Bacterial pneumonia (n=32, 44%) was more frequent than fungal (n=21, 29%) and viral pneumonia (n=14, 19%). The most frequent pathogens isolated in each group were: *Escherichia coli* (n=7, 9%), CMV (n=12, 15%), and *Aspergillus spp*. (n=12, 15%). Among bacteria, the most common aetiologies were *E. coli* and *P. aeruginosa*, as previously reported in other studies,^71^,^77^ whereas *S. pneumoniae* caused only 5% of pneumonias and this finding was possibly associated with the routine use of immunization and prophylaxis.

The median time of pneumonia diagnosis after transplantation was 66.5 days. Even if bacterial pneumonia is usually reported during the neutropenic phase soon after HSCT, in this study the pneumonias caused by Gram-negative bacilli appeared significantly later than pneumonia caused by moulds (p=0.02), possibly because *P. aeruginosa* pneumonia may occur later in the post-transplant period in patients developing GvHD.^77^

The global mortality rate in allogeneic HSCT recipients that had at least one pneumonia episode was 46% (n=44) compared to 13% (n=43) in those without any pneumonia episode (p<0.01; RR 3.37; 95%CI: 2.43–4.68). Clinical factors increasing the mortality rate in HSCT recipients developing a pulmonary complication were invasive fungal infection, acute or chronic GvHD, developing pneumonia in the first 100 days after transplantation, acute respiratory failure and septic shock.

The results of this prospective multicentre study confirm that pneumonia remains a frequent infectious complication after HSCT, contributing to significant mortality.

## Clostridium difficile infection

In last decade, there has been a growing interest in *Clostridium difficile* infection (CDI) because of the increasing rate of this infection. This epidemiological change has been ascribed to the emergence of an epidemic strain of *C. difficile* known as NAP-1, which has been associated with an increased frequency and severity of the disease. Nowadays CDI is the leading cause of infectious diarrhoea in hospitalized patients, and HSCT recipients appear to be one of the highest risk populations for this infection. In fact, Chopra et al. found that among all hospitalized patients in a non-outbreak setting, CDI rates in HSCT recipients were nine-fold higher than those in general patients and 1.4-fold higher than those in other patients with cancer.^78^ Therefore, a brief review of the available studies on CDI in HSCT recipients has been performed and is outlined in Table 2.

Referring to the reviewed literature, CDI affects between 5.7%^79^ and 24.7%^80^ of adult HSCT recipients during the first year after transplant, with the highest rates reported by the most recent studies. The same literature review showed that most CDI cases occur in the early post-transplant period with median time to diagnosis ranging between 3.5 days 80 and 33 days after HSCT (Table 2).^81^ Some authors observed that CDI is more likely to occur in the early phase of HSCT if recipients are pre-colonized with toxigenic *C. difficile*.^80^,^82^ Many studies reported high rates of infection due to NAP-1 strain, but Alonso et al. found that overall rates of CDI was not significantly different between the two centres involved in the study, despite differences in NAP-1 endemicity.^79^ Risk factors for CDI in hematopoietic transplant recipients are poorly understood. The difficulties in identifying unique risk factors for CDI in HSCT population may arise from the ubiquity of traditional risk factors for CDI in this population. In fact, most patients, if not all, receive broad spectrum antibiotics, have a prolonged hospital stay, have an altered integrity of the intestinal mucosa, and all are severely ill and immunocompromised. Furthermore, the use of allogeneic HSCT has expanded progressively to older patients due to the development of reduced intensity conditioning regimens. Thus, both older age and the presence of comorbidities are increasingly frequent in HSCT setting.

Many studies focused on risk factors for CDI in HSCT population, and several risk factors have been identified. They are reported in Table 3. Some authors found that CDI occurred significantly more often in allogeneic recipients (incidence 12.5%–21.3%) than in the autologous recipients (incidence 5.7%–9.2%).^78^,^83^ On the contrary, a recent prospective study by Bruminhent et al. showed no difference in the incidence of CDI in patients receiving autologous and allogeneic HSCTs (24% versus 25%, respectively).^80^ Other possible risk factors for developing CDI are use of broad-spectrum antimicrobials,^79^,^81^,^82^,^84^ and acute GvHD,^81^,^85^ while myeloablative conditioning regimen increased the risk in some,^81^,^82^,^85^ but not all cohorts.^86^ The only variable associated with a reduced risk of CDI was the use of growth factors.^84^ Interestingly, Bruminhent et al. analysed the relationship between prior *C. difficile* colonization and CDI. In this prospective study at least 10.7% of patients admitted for HSCT were colonized with a toxigenic strain and nearly all of them (87.5%) developed CDI, compared to 17.2% of patients with negative colonization status at hospital admission (p < 0.01).^80^

The most controversial issue is a potentially important interplay between CDI and gastrointestinal GvHD. Whereas some studies showed a strong relationship between early CDI and subsequent development of gastrointestinal GvHD in the first year following allogeneic HSCT,^81^,^84^,^87^ this association has not been confirmed by other studies.^80^,^82^,^85^,^86^ As far as clinical course of CDI is concerned, the disease in most studies was uniformly mild, irrespective of the rate of infections due to NAP-1 strain,^86^ and no differences in the mortality rates were observed in patients with or without CDI.^78^,^79^,^81^,^82^,^85^ The low percentage of complications in this patients population may be due to a decreased inflammation from immunosuppression related to transplantation.^79^ In fact, only one study found that HSCT recipients with CDI were more likely to develop GvHD, BSIs and had lower survival rate when compared to controls.^84^ In contrast to other studies reporting little impact of CDI on mortality in HSCT recipients, a recent Brazilian experience of 64 patients with CDI, including 31 cases after allogeneic and 14 after autologous HSCT, demonstrated a significant impact of CDI on survival. In particular, a severe form of CDI developed in 23% of allogeneic HSCT recipients, and all of them died.^88^ Of note, 89% of patients in this cohort received initial treatment with metronidazole that might have influenced the clinical course of CDI.

One of the main problems of CDI in the immunocompromised is a high rate or recurrent infections. In fact, in HSCT recipients recurrence rates ranged between 2.6%^85^ and 31%,^86^ and they were more frequent in those patients who received metronidazole monotherapy compared to those who received vancomycin-containing regimens.^81^ Other risk factors for recurrent disease were neutropenia at the onset of CDI^8^ and infection due to NAP-1 strain.^86^ The frequent use of proton pump inhibits might also contribute to recurrences, as recently demonstrated in a general patient population.^89^

The management of CDI is based on prompt diagnosis, effective treatment and strict application of contact precautions which do not differ between HSCT recipients and other vulnerable.^90^

In conclusion, CDI is one of the most frequent causes of infectious diarrhoea in HSCT recipients, and it occurs early in the post-transplant period. Updated diagnostic and treatment algorithms for CDI should be put in place. Since many of the risk factors for CDI are not easily modifiable in this population, the predisposing role of pre-transplant colonization with *C. difficile* warrants further studies. Although CDI represent an important cause of morbidity for this population, non-severe forms of CDI are predominant, and associated mortality seems low.

### Recent advances in the management of bacterial infections

Since any delay in starting an effective antibiotic therapy for the treatment of bacterial infections (particularly due to Gram-negatives) has been associated with an increased mortality, empirical therapy directed against Enterobacteriaceae and *P. aeruginosa* has been a cornerstone of managing bacterial infections during neutropenia for decades.^1^ Ceftazidime, cefepime, piperacillin/tazobactam or carbapenems are listed as suitable options.^1^

The only recent trial on empirical therapy reported on the use of oral moxifloxacin in low risk patients with febrile neutropenia, and found it non inferior to the standard oral option of amoxicillin/clavulanate and ciprofloxacin.^91^ However, this novel regimen is unsuitable for HSCT recipients since they are usually high risk patients and frequently receive fluoroquinolone prophylaxis during neutropenia.

In the times when resistant pathogens are seen on a daily basis in many centres, the main advance in the management of bacterial infections in HSCT is a novel individualised approach to the empirical antibiotic therapy.^92^ In fact, ECIL-4 recommendations on the empirical therapy of febrile neutropenia propose two different approaches based on clinical presentation and the risk for infection due to a resistant strain.^92^ The classical escalation strategy is defined as starting an antibiotic which covers susceptible Enterobacteriaceae and *P. aeruginosa*, but not ESBL-producers, carbapenem-resistant *K. pneumoniae* or other MDR strains. Then, if patient’s clinical conditions deteriorate, or if a resistant pathogen is isolated, therapy is escalated to cover suspected or isolated resistant bacteria. Its advantages include: 1) limiting early use of a combination therapy or a broadest spectrum antibacterial, such as carbapenem, 2) low toxicity, 3) usually lower costs, and 4) hopefully, less selection of resistant strains. Anti-pseudomonal cephalosporins, such as cefepime or ceftazidime, or piperacillin/tazobactam are the most frequently used treatment options. The novelty in approaching empirical antibiotic therapy in neutropenia consists of introducing a strategy that has been used widely so far in the intensive care unit setting. De-escalation approach means starting upfront a regimen covering the most dangerous resistant pathogens, i.e. ESBL-producers, MDR P. aeruginosa etc.^92^ The main point of using a de-escalation strategy is to start active treatment of a suspected resistant Gram-negative, hopefully resulting in reduced mortality. Its main limit is a frequently unnecessary routine use of broad spectrum molecules or a combination therapy with nephrotoxic agents such as aminoglycosides or colistin.

The most difficult clinical decision is establishing which patients might benefit from a de-escalation approach and which may still be confidently treated with a classical escalation approach. From the review of the literature and personal experience, the most frequent risk factors for infection with resistant bacteria are: prior infection or colonisation with a non-susceptible strain and being admitted to or coming from a centre where resistant bacteria are frequent.^92^ De-escalation treatment is usually administered to subjects with one of the aforementioned risk factors who develop sepsis or septic shock during neutropenia. The management of infections caused by antibiotic resistant Gram-negative bacteria in HSCT recipients has been recently reviewed.^93^

### Infection control measures

Non-pharmacological management of bacterial infections is of outmost importance in the era of increasing bacterial resistance. It includes screening for resistant bacterial and applying infection control measures in case of transmissible pathogens. Of note, these include not only MDR Gram-negatives or VRE, but also *C. difficile*. Hand hygiene and contact precautions (gloves and gown) are the most effective infection control strategies that apply to the prevention of the spread of any pathogens.

Surveillance cultures for MDR bacteria identify patients colonised with resistant strains. This knowledge, not only allows to avoid actively transmission to other HSCT recipients by applying contact precautions, but may also suggest which antibiotics might be appropriate for empirical treatment. Another theoretical possibility is to pursuit decontamination of the colonised patients, although the data on decolonisation in HSCT setting are almost inexistent, and the results are far from promising. Additionally, the risk of inducing resistance to the last available treatment option in case of MDR Gram-negative rods should be counterbalance with an evident long-term benefit of decontamination.^93^

### Antimicrobial stewardship

Last but not least, the management of bacterial infections in HSCT should include a formal program on antimicrobial stewardship.^94^ Its main objectives are to improve the outcome of infections and to reduce inappropriate use of antimicrobials (e.g. discontinue if not necessary, promote the use of correct dosage). Additional aims include reducing side effects of antibiotic therapies, i.e. direct toxicity or influence on local epidemiology, and hopefully, but not automatically, reducing costs of antibiotic treatments (by withholding antibiotic treatment if not necessary, de-escalation to narrow spectrum agents if possible, etc.).

Running a successful antimicrobial stewardship program is based on a multidisciplinary approach, with a dedicated team that includes, among others, infectious diseases specialist, microbiologist, clinical pharmacologist and infection control specialist, and on approval and endorsement of hospital authorities, which enable to allocate necessary resources.

One of important points of reviewing antibiotic prescriptions are clinical audits to identify the critical areas for antibiotic use in HSCT unit (e.g. inappropriate indications, incorrect dosage, routine prescriptions off-label, too long therapies, no intravenous to oral switch, etc.) and a thorough knowledge of local epidemiology of the most frequent pathogens, the rate of resistance to various antimicrobials, and clinical outcome of these infections.

### Conclusions

Bacterial infections continue to be one of the most frequent complications after HSCT. The incidence of Gram-negative bacteria and the rate of resistance to antibiotics have been steadily increasing in many centres. However, important differences in the epidemiology of bacterial infections exist among transplant centres worldwide. Therefore, the knowledge of local epidemiology is crucial and should guide the approach to antibiotic prophylaxis, empirical therapy and management of infections. Numerous interesting issues such as the role of surveillance cultures for guiding empirical therapy, the benefits of protocols for screening for resistant bacteria, decolonisation and the current role of antibiotic prophylaxis in HSCT setting await to be addressed in future clinical studies.

## Figures and Tables

**Table 1:** The main risk factors associated with BSI due to single bacterial species

Risk factor	Bacterial species
Oral mucositis	Viridans streptococci Coagulase-negative staphylococci
Enteric mucositis	Enterobacteriaceae Enterococci *Pseudomonas aeruginosa*
Extensive and prolonged use of central venous catheters	Staphylococci
Lower performance status/comorbidities	Enterococci
Graft-versus-Host Disease	Gram/negative bacteria, including MDR *P. aeruginosa*
Graft-versus-Host Disease Hypogammaglobulinemia	Pneumococci
Fluoroquinolone prophylaxis	Staphylococci Enterococci Viridans streptococci
Use of cephalosporins	Enterococci, viridans streptococci (ceftazidime)
Treatment with beta-lactams	Beta-lactam resistant viridans streptococci
Nasal colonisation due to MRSA	MRSA
Colonisation with VRE	VRE

MRSA, methicillin-resistant *Staphylococcus aureus*; VRE, vancomycin-resistant enterococci.

**Table 2:** Studies evaluating *Clostridium difficile* infection in HSCT recipients, 2010 to present.

Author (year)	Study period	HSCT type	Patients, no.	Rate of CDI	Median time to diagnosis after HSCT in days	Rate of recurrence
**Chopra et al. (2011)**	2005–2006	Both	361	14% (Both); 8% (Auto); 18% (Allo)	/	0% (Auto); 5% (Allo)
**Willems et al. (2012)**	2004–2007	Allo	407	13% (Allo)	25	2,6% (Allo)
**Alonso et al. (2012)**	2003–2008	Both	999	9,2% (Both); 6,5% (Auto); 12,5% (Allo)	6,5 (Auto); 33 (Allo)	21,7% (Both)
**Kamboj et al. (2012)**	/	Both (adult and paediatric patients)	/	9% (Auto); 27% (Allo)	/	/
**Trifilio et al. (2013)**	2004–2008	Both	822	10,3% (Both)	8	12% (Both)
**Alonso et al. (2013)**	2003–2008	Autologous	873	6% (Auto)	11	15,4% (Auto)
**Kamboj et al. (2014)**	2005–2010	Allogeneic (adult and paediatric patients)	793	21,3% (Allo)	/	31% (Allo)
**Kinnebrew et al. (2014)**	2009–2011	allo	94	17% (Allo)	/	/
**Huang et al. (2014)**	2010–2012	both	711	13,4% (Both); 9,2% (Auto); 18,2% (Allo)	/	22,9% (Auto); 23,3% (Allo)
**Bruminhent et al. (2014)**	2011–2012	both	150	24,7% (Both); 24,1% (Auto); 25% (Allo)	3,5	8.1% (Both)

Allo, Allogeneic HSCT; Auto, Autologous; CDI, *Clostridium difficile* infection.

**Table 3:** Risk factors for developing *Clostridium difficile* infection in HSCT recipients.

Risk factors	Reference
Allogeneic transplant	78,87
Cord blood transplant	85
Age > 60 years	87
Diabetes	84
Myeloablative conditioning regimen	81,82,85
Pre-engraftment period	84
*Clostridium difficile* colonization	80,82
Colonization with vancomycin-resistant enterococci	81,87
Severe mucositis (>= 2 grade)	79
Broad spectrum antibiotics	79,81,82,84
Graft-versus-Host Disease	81,85
